# Nucleotide sequence alignment of *hdcA* from Gram-positive bacteria

**DOI:** 10.1016/j.dib.2016.01.020

**Published:** 2016-01-18

**Authors:** Maria Diaz, Victor Ladero, Begoña Redruello, Esther Sanchez-Llana, Beatriz del Rio, Maria Fernandez, Maria Cruz Martin, Miguel A. Alvarez

**Affiliations:** Instituto de Productos Lácteos de Asturias, IPLA-CSIC, Paseo Rio Linares s/n, 33300 Villaviciosa, Spain

## Abstract

The decarboxylation of histidine -carried out mainly by some gram-positive bacteria- yields the toxic dietary biogenic amine histamine (Ladero et al. 2010 〈10.2174/157340110791233256〉 [1], Linares et al. 2016 〈http://dx.doi.org/10.1016/j.foodchem.2015.11.013〉〉 [2]). The reaction is catalyzed by a pyruvoyl-dependent histidine decarboxylase (Linares et al. 2011 〈10.1080/10408398.2011.582813〉 [3]), which is encoded by the gene *hdcA*. In order to locate conserved regions in the *hdcA* gene of Gram-positive bacteria, this article provides a nucleotide sequence alignment of all the *hdcA* sequences from Gram-positive bacteria present in databases. For further utility and discussion, see 〈http://dx.doi.org/ 10.1016/j.foodcont.2015.11.035〉〉 [4].

**Specifications table**

**Table:** 

Subject area	*Microbiology*
More specific subject area	*Food microbiology*
Type of data	*Figure*
How data was acquired	*Retrieved from databases*
Data format	*Analyzed*
Experimental factors	*The nucleotide sequences were retrieved from NCBI database shown below*
Experimental features	*Sequences were aligned using ClustalW software and visualized using Jalview v.2 programme.*
Data source location	*N/A*
Data accessibility	*Data with article*

**Value of the data**

• Identification of Gram-positive bacteria *hdcA* gene present at databases.
• Sequence alignment defines conserved and variable regions within the *hdcA* sequence.
• Obtained data allowed to design primers within *hdcA* conserved regions -flanking variable ones- that can be used to identify histamine-producing species in food matrices by PCR-DGGE.

## 1 Data

**Fig. 1:**
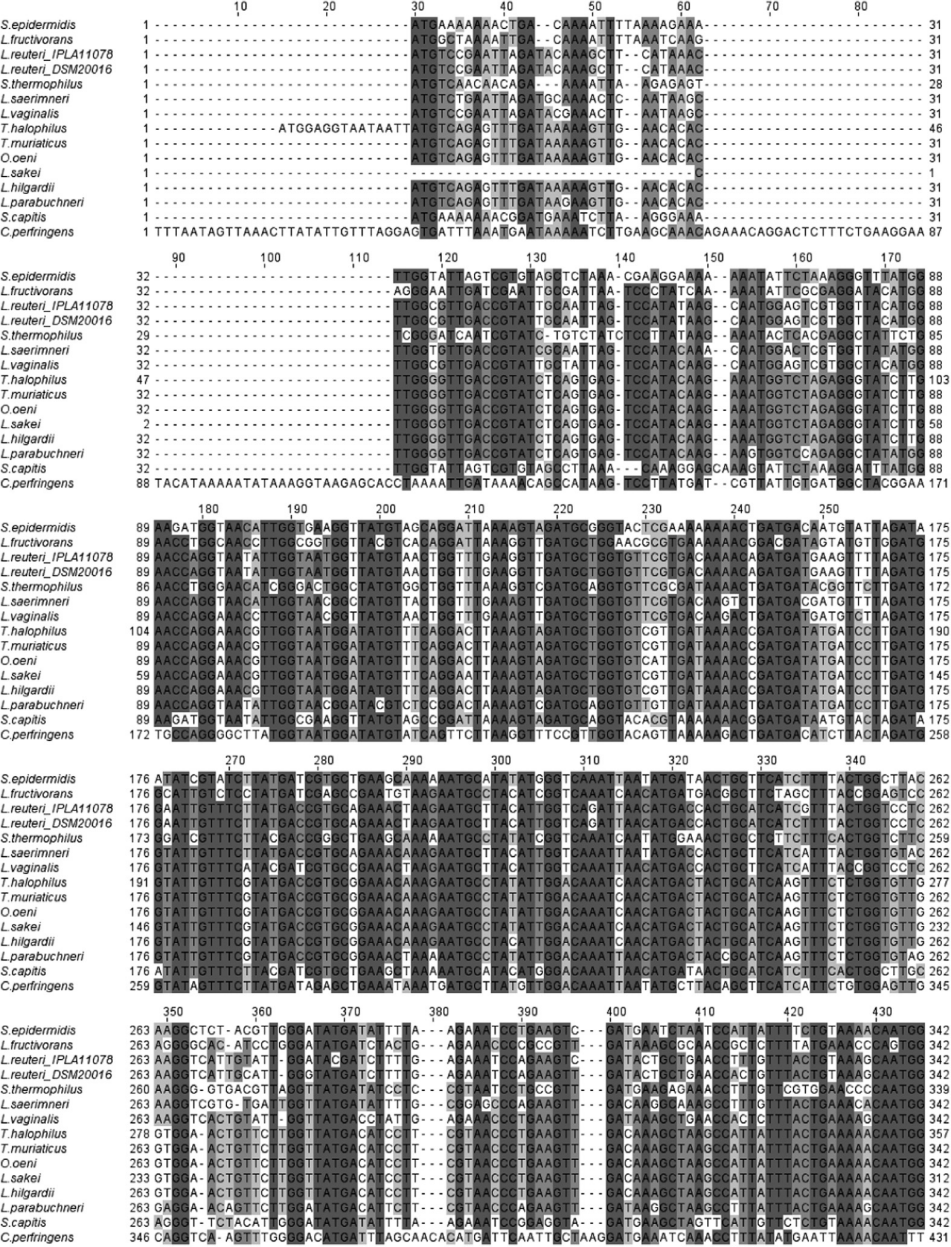

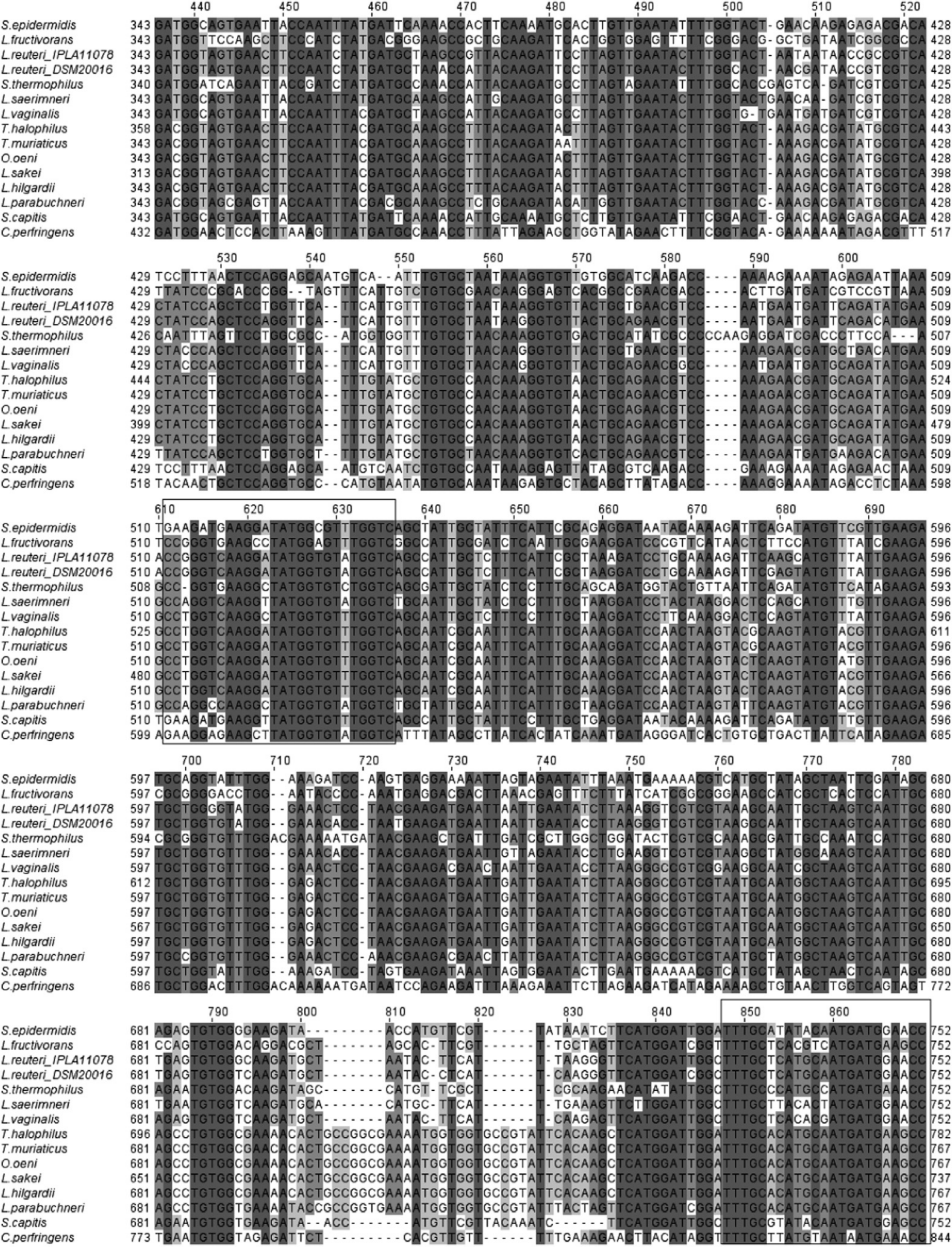

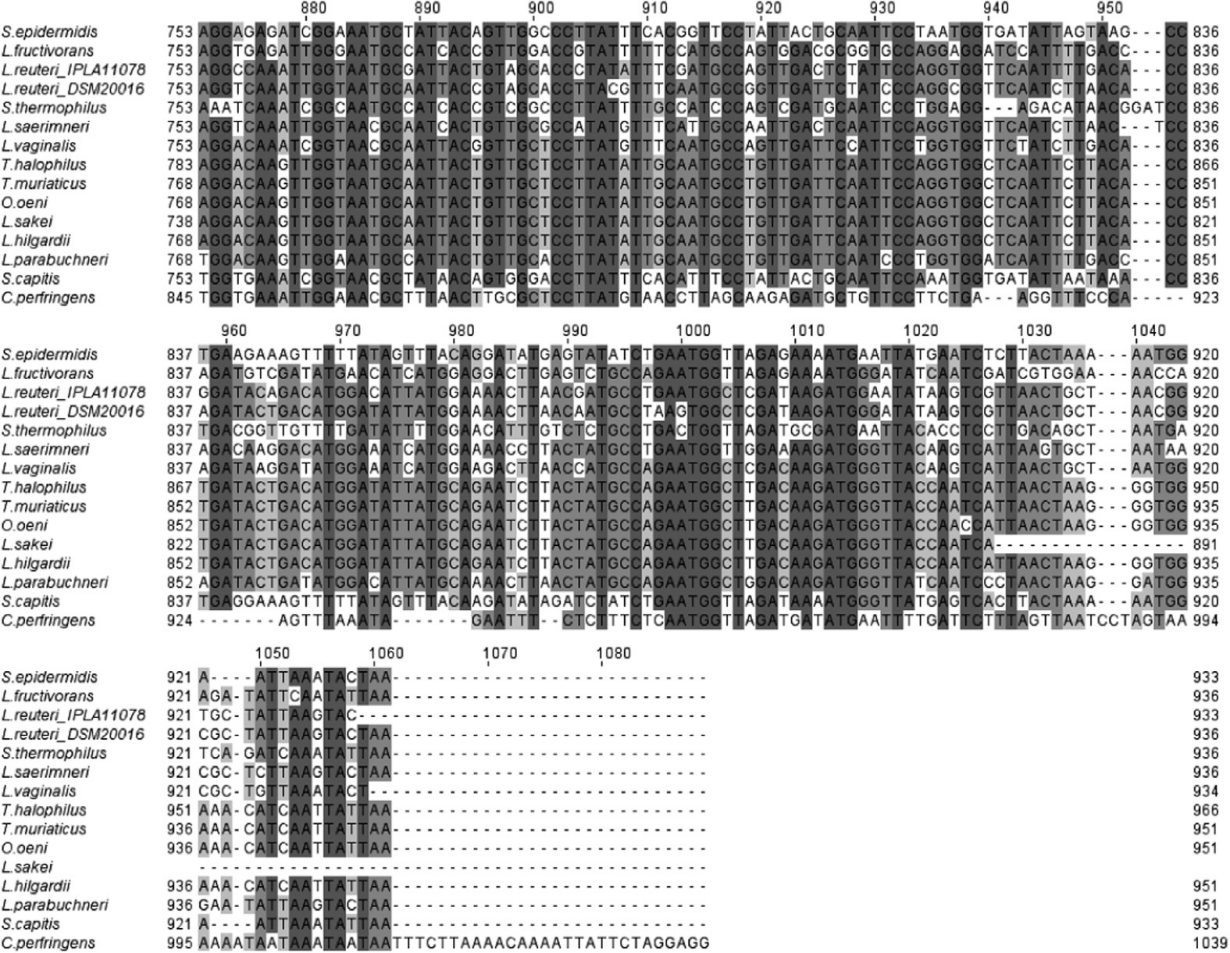
*hdcA* sequences of Gram-positive bacteria strains, which are indicated in Table 1, were aligned using ClustalW software. The bacterial species are indicated at the left of each sequence. The gray tones correspond to the percentage of residues in each column that agree with a hypothetical consensus sequence: >80%, >60%, >40%, <40%. Selected regions for primer design are indicated with a box.

Full-length *hdcA* sequences of the histamine-producing Gram-positive bacteria strains present in Genbank database were aligned using ClustalW software [5]. Based on the alignment of these sequences Fig. 1 was made and primers used in Diaz et al. 2016 [4] were designed. For reasoning and further utility and discussion, see [1], [2], [3], [4].

## 2 Experimental design, materials and methods

Sequence data were obtained from NCBI (http://www.ncbi.nlm.nih.gov/) database. Full-length *hdcA* sequences of representative histamine-producing Gram-positive bacteria strains present in database were selected for the alignment (Table 1). The sequences were aligned by DNA multiple sequence alignment ClustalW software using default parameters [5]. The visualization, which included the percentage of conservation of each residue was obtained using Jalview v.2 programme [6].

## Figures and Tables

**Table 1:** Accession numbers of the *hdcA* gene sequences of representative histamine-producing Gram-positive bacteria strains present in database used for the alignment.

***hdc+*****species**	**Accession number**
*Staphylococcus epidermidis*	GeneBank: AB583189
*Lactobacillus fructivorans*	GeneBank: NZ_JOJZ01000009
*Lactobacillus reuteri* IPLA11078	GeneBank: LN877767
*L. reuteri* DSM20016	GeneBank: NC009513
*Streptococcus thermophilus*	GeneBank: FN686789
*Lactobacillus saerimneri* 30a	GeneBank: NZ_ANAG0000000
*Lactobacillus vaginalis*	GeneBank: LN828720
*Tetragenococcus halophilus*	GeneBank: AB362339
*Tetragenococcus muriaticus*	GeneBank: DQ132889
*Oenococcus oeni*	GeneBank: DQ132887
*Lactobacillus sakei*	GeneBank: DQ132888
*Lactobacillus hilgardii*	GeneBank: AY651779
*Lactobacillus parabuchneri*	GeneBank: LN877764
*Staphylococcus capitis*	GeneBank: AM283479
*Clostridium perfringens*	GeneBank: BA000016
